# Crystal structures, syntheses, and spectroscopic and electrochemical measurements of two push–pull chromophores: 2-[4-(di­methyl­amino)­benzyl­idene]-1*H*-indene-1,3(2*H*)-dione and (*E*)-2-{3-[4-(di­meth­ylamino)­phen­yl]allyl­idene}-1*H*-indene-1,3(2*H*)-dione

**DOI:** 10.1107/S205698901901329X

**Published:** 2019-10-03

**Authors:** Georgii Bogdanov, John P. Tillotson, Tatiana Timofeeva

**Affiliations:** a Department of Chemistry, New Mexico Highlands University, Las Vegas, New Mexico, 87701, USA; b School of Chemistry and Biochemistry, Georgia Institute of Technology, Atlanta, Georgia, 30332, USA

**Keywords:** crystal structure, donor–π-bridge–acceptor, non-linear chromophores, indane derivatives, NLO

## Abstract

The title pull–push chromophores, 2-[4-(di­methyl­amino)­benzyl­idene]-1*H*-indene-1,3(2*H*)-dione, C_18_H_15_NO_2_ (ID[1]) and (*E*)-2-{3-[4-(di­methyl­amino)­phen­yl]allyl­idene}-1*H*-indene-1,3(2*H*)-dione, C_20_H_17_NO_2_ (ID[2]), have donor–π-bridge–acceptor structures. The mol­ecule with the short π-bridge, ID[1], is almost planar while for the mol­ecule with a longer bridge, ID[2], is less planar. The benzene ring is inclined to the mean plane of the 2,3-di­hydro-1*H*-indene unit by 3.19 (4)° in ID[1] and 13.06 (8)° in ID[2]. The structures of three polymorphs of compound ID[1] have been reported: the α-polymorph [space group *P*2_1_/*c*; Magomedova & Zvonkova (1978 ▸). *Kristallografiya*, **23**, 281–288], the β-polymorph [space group *P*2_1_/*c*; Magomedova & Zvonkova (1980 ▸). *Kristallografiya*, **25** 1183–1187] and the γ-polymorph [space group *Pna*2_1_; Magomedova, Neigauz, Zvonkova & Novakovskaya (1980 ▸). *Kristallografiya*, **25**, 400–402]. The mol­ecular packing in ID[1] studied here is centrosymmetric (space group *P*2_1_/c) and corresponds to the β-polymorph structure. The mol­ecular packing in ID[2] is non-centrosymmetric (space group *P*2_1_), which suggests potential NLO properties for this crystalline material. In both compounds, there is short intra­molecular C—H⋯O contact present, enclosing an *S*(7) ring motif. In the crystal of ID[1], mol­ecules are linked by C—H⋯O hydrogen bonds and C—H⋯π inter­actions, forming layers parallel to the *bc* plane. In the crystal of ID[2], mol­ecules are liked by C—H⋯O hydrogen bonds to form 2_1_ helices propagating along the *b-*axis direction. The mol­ecules in the helix are linked by offset π–π inter­actions with, for example, a centroid–centroid distance of 3.9664 (13) Å (= *b* axis) separating the indene rings, and an offset of 1.869 Å. Spectroscopic and electrochemical measurements show the ability of these compounds to easily transfer electrons through the π-conjugated chain.

## Abstract 2

The title pull–push chromophores, 2-[4-(di­methyl­amino)­benzyl­idene]-1*H*-indene-1,3(2*H*)-dione (ID[1]) and (*E*)-2-{3-[4-(di­methyl­amino)­phen­yl]allyl­idene}-1*H*-indene-1,3(2*H*)-dione (ID[2]), with donor–π-bridge–acceptor structures, are almost planar for the mol­ecule with a short π-bridge (ID[1]) but less planar for the mol­ecule with a longer bridge (ID[2]).

## Chemical context   

Organic mol­ecules containing donor and acceptor groups connected by a conjugated π-bridge (push–pull chromophores) are important in many areas of materials chemistry, especially organic electronics and optoelectronics. Applic­ations of pull–push mol­ecules can be related to their properties such as intra­molecular charge transfer and specific mol­ecular arrangements in the solid state. Intra­molecular charge transfer from donor to acceptor *via* a π-bridge defines their colour, light absorption and emission, hyperpolarizability and other optoelectronic effects. Applications of pull–push chromophores include non-linear optics (NLO; Ortiz *et al.*, 1994 ▸), as luminescent sensors (Duarte *et al.*, 2011 ▸; Qin *et al.*, 2015 ▸), solid-state lasers (Samuel & Turnbull, 2007 ▸), organic light-emitting diodes (Muller *et al.*, 2003 ▸), organic field-effect transistors (Suponitsky *et al.*, 2006 ▸; Oliveira *et al.*, 2018 ▸) and many more. The spectroscopic properties of pull–push mol­ecules are related to the donor and acceptor strength in these mol­ecules and to the length of the π-bridge. Many such compounds have been studied, but not all of their crystal structures have been reported. Such compounds are important for their NLO properties (Andreu *et al.*, 2003 ▸; Raimundo *et al.*, 2002 ▸). Herein, we report on the crystal structures, syntheses and spectroscopic and electrochemical properties of the title donor–π-bridge–acceptor structures, ID[1] and ID[2]. The structures of three polymorphs of ID[1] have been reported previously; the α-polymorph (Magomedova & Zvonkova, 1978 ▸), the β-polymorph (Magomedova & Zvonkova, 1980 ▸) and the γ-polymorph (Magomedova, Neigauz *et al.*, 1980 ▸). We have repeated the structural study of ID[1] in order to establish exactly which polymorph we obtained. It was then characterized by spectroscopic and electrochemical measurements.
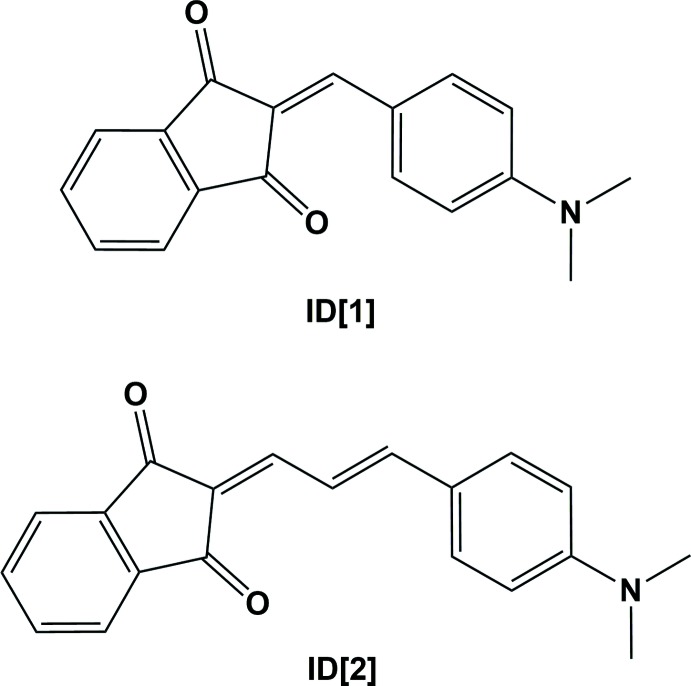


## Structural commentary   

**Figure 1:**
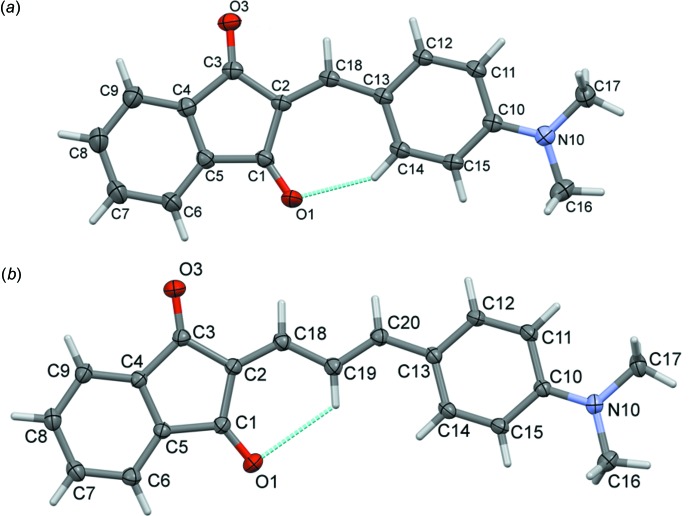
A view of the mol­ecular structure of the title compounds, (*a*) ID[1] and (*b*) ID[2], with atom labelling. Displacement ellipsoids are drawn at the 50% probability level. The intra­molecular C—H⋯O hydrogen bonds (Tables 2 ▸ and 3 ▸) are shown as dashed lines.

The mol­ecular structures of ID[1] and ID[2] are illustrated in Fig. 1 ▸. The structural analysis of ID[1] synthesized by us established that it is the β-polymorph (Magomedova & Zvonkova, 1980 ▸), and it was then characterized with spectroscopic (§4) and electrochemical (§5) measurements.

Both mol­ecules have acceptor–π-bridge–donor structures. It was found, as in our previous studies (Tillotson *et al.*, 2019 ▸), that with an increase of the length of the π-conjugated bridge the mol­ecule becomes less planar, and the angles between the different planar fragments (acceptor–bridge, bridge–donor) become larger (Table 1 ▸).

Compound ID[1] has an almost planar structure, with the benzene ring (C10–C15) being inclined to the mean plane of the indene ring system (C1–C9) by 3.19 (4)°. In ID[2] the deviation from planarity is somewhat larger with the benzene ring (C10–C15) being inclined to the mean plane of the indene ring system (C1–C9) by 13.06 (8)°; see further details in Table 1 ▸.

## Supra­molecular features   

Mol­ecules of ID[1] and ID[2] have significant dipole moments, which is very common for NLO chromophores. Because of this, mol­ecules have a trend to anti­parallel packing, which is observed in the crystal structures of both ID[1] and ID[2].

**Figure 2:**
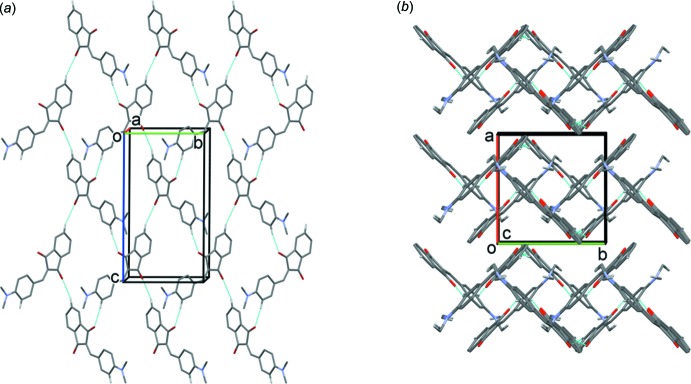
Views along (*a*) the *a* axis and (*b*) the *c* axis of the crystal packing of ID[1]. The hydrogen bonds (Table 2 ▸) are shown as dashed lines. For clarity, only the H atoms involved in the inter­molecular inter­actions have been included.

In the crystal of ID[1], mol­ecules form two almost perpendicular stacks with anangle of *ca* 84.47° between them. The mol­ecules, which stack in an anti­parallel or head-to-tail fashion, are linked by C—H⋯O hydrogen bonds (Fig. 2 ▸, Table 2 ▸), forming layers lying parallel to the *bc* plane. Within the layers there are C—H⋯π inter­actions present (Table 2 ▸).

**Figure 3:**
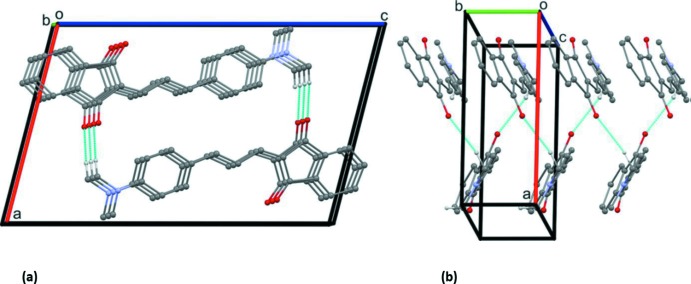
Views along (*a*) the *b* axis and (*b*) the *c* axis of the crystal packing of ID[2]. The hydrogen bonds (Table 3 ▸) are shown as dashed lines. For clarity, only the H atoms involved in the inter­molecular inter­actions have been included.

In the crystal of ID[2], mol­ecules form stacks with parallel mol­ecular positions, and shifted positions of stacks extended along the *b-*axis direction within the acentric space group *P*2_1_. The ID[2] mol­ecules are packed in a herringbone fashion (Fig. 3 ▸). Here, the angle between two mol­ecules from different stacks is *ca* 60.8°. The mol­ecules are linked by C—H⋯O hydrogen bonds (Table 3 ▸), forming a 2_1_ helix that propagates along the *b*-axis direction. The mol­ecules in the helix are linked by offset π–π inter­actions with, for example, a centroid–centroid distance *Cg*1⋯*Cg*1^i^ of 3.9664 (13) Å [symmetry code: (i) *x*, *y* − 1, *z*] separating the indene ring systems (C1–C9), with an offset of 1.869 Å.

## Spectroscopic studies   

**Figure 4:**
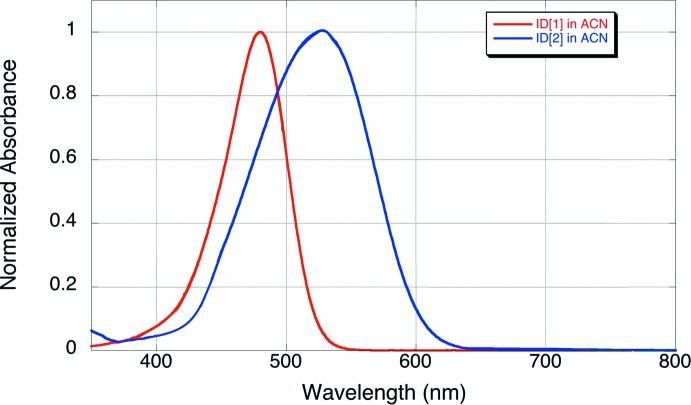
Normalized absorbance spectra (nm) in aceto­nitrile for ID[1] and ID[2].

Absorbance spectra were obtained for both ID[1] and ID[2] in chloro­form and aceto­nitrile. For donor–acceptor polyenes, the dominating feature of the absorbance spectrum is the π–π* transition that results from charge transfer from donor to acceptor. According to recent studies (Bogdanov *et al.*, 2019 ▸), it proves that di­methyl­amino­phenyl polyenals have reversed solvatochromism, which is proved by the maxima of the absorption values (Table 4 ▸), showing that both ID[1] and ID[2] have their peaks higher in chloro­form than in aceto­nitrile; see the absorption spectra of ID[1] and ID[2] in aceto­nitrile given in Fig. 4 ▸.

## Electrochemical measurements   

**Figure 5:**
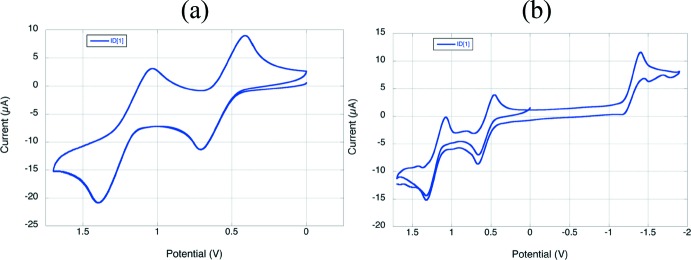
Cyclic voltammagrams of ID[1]: (*a*) sweep from 0 to 1.7 V and (*b*) sweep from −1.9 to 1.7 V.

Donor–acceptor polyenes can be characterized by electrochemical measurements to show their ability to transfer electrons. The voltammagrams (Fig. 5 ▸) demonstrate a completely reversible oxidation process and a partially reversible reduction process. When only swept between 0 V and 1.7 V the oxidation process is reversible (Fig. 5 ▸ *a*), however, when swept to −1.9 V the reduction is only partly reversible (Fig. 5 ▸ *b*). This represents the ability of the compound to ‘easily’ transfer electrons through the chain from donor towards acceptor.

Note: cyclic voltammagrams of ID[1] were made against FeCp_2_^+/0^ (inter­nal reference E_1/2_^+/0^ = 0.55 V *vs* Ag/AgCl) in di­chloro­methane with 0.1 *M ^n^*Bu_4_NPF_6_). Measurements were recorded at 50 mV s^−1^ using a BAS Potentiostat using a glassy carbon working electrode, Pt wire auxilliary electrode and a Ag/AgCl reference electrode.

## Database survey   

A search of the Cambridge Structural Database (CSD Version 5.40, update May 2019; Groom *et al.*, 2016 ▸) for the substructure of ID[1] yielded 27 hits. Three of them, 2-(*p*-di­ethyl­amino­benzyl­idene)-1,3-indandione (CSD refcode TELWEM; Khodorkovsky *et al.*, 1996 ▸), which has ethyl groups instead of methyl groups in the donor, 2-{[4-(di­phenyl­amino)­phen­yl]methyl­idene}-1*H*-indene-1,3(2*H*)-dione (QENYEQ at 223 K: Hariharan *et al.*, 2018 ▸; QENYEQ01 at 150 K: Redon *et al.*, 2018 ▸), which has methyl groups in the donor, and 2-{[4-(di­butyl­amino)­phen­yl]methyl­idene}-1*H*-indene-1,3(2*H*)-di­one (BIQYUY; Situ *et al.*, 2019 ▸), which has butyl groups in the donor. In these three compounds, the benzene ring is inclined to the mean plane of the 2,3-di­hydro-1*H*-indene ring system by 7.6 (2), 1.66 (6)/1.49 (9) and 5.71 (9)°, for TELWEN, QENYEQ/QENYEQ01 and BIQYUY, respectively, compared to 3.19 (4)° in ID[1].

Also, out of all 27 hits there are three hits, (MBYINO: Magomedova *et al.*, 1978 ▸; MBYINO01: Magomedova, Neigauz *et al.*, 1980 ▸; MBYINO02: Magomedova & Zvonkova, 1980 ▸), which are the α, γ and β ID[1] polymorphs, respectively, published over 40 years ago. It should be mentioned that the crystal packing in the α and β polymorphs is centric (space group *P*2_1_/*c*), while in the γ polymorph it is acentric (space group *Pna*2_1_). The crystal structure of ID[1] we obtained corresponds to the β polymorph, *i.e*. the centrosymmetric modification MBYINO02. The dihedral angles between the benzene and indene rings for two independent mol­ecules in MBYINO are *ca* 4.35 and 7.79°, compared to *ca* 7.36° in MBYINO01, and 3.54° in MBYINO02 (*cf*. 3.19 (4)° in the present crystal structure analysis of ID[1]).

A search of the CSD for the substructure of ID[2] yielded nine hits. Only two structures are similar to that of ID[2]. The first, 2-{3-[4-(di­methyl­amino)­phen­yl]prop-2-yn-1-yl­idene}-1*H*-indene-1,3(2*H*)-dione (ZIGPIR; Solanke *et al.*, 2018 ▸), has a triple bond between atoms C18 and C19. The second, 2-[4-(di­methyl­amino)­cinnamo­yl]indan-1,3-dione (CNINDO; Magomedova, Zvonkova *et al.*, 1980 ▸) has a hydroxyl group attached to atom C18. Like ID[2], it crystallizes in the chiral monoclinic space group *P*2_1_. The benzene ring is inclined to the mean plane of the indene ring system by *ca* 11.42° in CNINDO compared to 13.06 (8)° in ID[2]. In ZIGPIR this dihedral angle is smaller at 8.6 (3)°.

## Synthesis and crystallization   

For the synthesis of the title compounds, two aldehydes were used: 4-(di­methyl­amino)­benzaldehyde (A1; purchased from Aldrich) and 4-(di­methyl­amino)­cinnamaldehyde (A2), which was synthesized as described previously (Tillotson, *et al.*, 2019 ▸). 2,3-Indanedione was purchased from Aldrich and used without further purification.

**Synthesis of 2-[4-(di­methyl­amino)­benzyl­idene]indane-1,3-dione (ID[1]):** Aldehyde A1 (2.00 g, 13.4 mmol) and 1,3-indanedione (2.01 g, 13.4 mmol) were suspended in 100 ml of absolute ethanol. The mixture was gently heated until the solids had dissolved. After about 10 min of stirring the dissolution was complete, and a red crystalline precipitate began forming on the walls of the flask. The reaction mixture was stirred vigorously overnight, and the resulting product was collected by filtration then washed with cold ethanol and hexa­nes to give shiny dark-red crystals (yield 3.65 g, 98%; m.p. 477–478 K). ID[1] can be purified by recrystallization using numerous solvent systems (acetone, ethanol, ethyl acetate/hexane, di­chloro­methane/hexane and toluene (to name a few), many of which afforded single crystals. ^1^H NMR (400 MHz, CD_2_Cl_2_) δ 8.52 (*d*, *J* = 9.16 Hz, 2H), 7.91–9.73 (*m*, 4H), 7.71 (*s*, 1H), 6.77 (*d*, *J* = 9.16 HZ, 2H), 3.14 (*s*, 6H) ppm. ^13^C NMR (100 MHz, CD_2_Cl_2_) δ 191.6, 190.1, 154.5, 147.3, 142.7, 140.3, 138.2, 134.8, 134.5, 123.3, 122.7, 122.6, 122.2, 111.7, 40.3 ppm.

**Synthesis of (** ***E*** **)-2-{3-[4-(di­methyl­amino)­phen­yl]allyl­idene}indane-1,3-dione (ID[2]):** Aldehyde A2 (1.00 g, 5.71 mmol), 1,3-indanedione (0.85 g, 5.7 mmol) and piperidine (0.15 ml, 1.4 mmol) in ethanol (50 ml) were mixed and treated as for the synthesis of ID[1]. The crude product obtained was collected by filtration and washed with cold ethanol before being recrystallized from ethanol to give incredibly shiny and thin purple actinic crystals (1.55 g, 90%; m.p. 535–537 K). They were washed with hexane and dried under vacuum. ^1^H NMR (400 MHz, CD_2_Cl_2_) δ 8.25 (*dd*, *J* = 15.0, 12.2 Hz, 1H), 7.90–7.86 (*m*, 2H), 7.76–7.73 (*m*, 2H), 7.60 (*d*, *J* = 12.2 Hz, 1H), 7.60 (*d*, *J* = 9.0 Hz, 2H), 7.34 (*d*, *J* = 15.0 Hz, 1H), 6.72 (*d*, *J* = 9.0 Hz, 2H), 3.08 (*s*, 6H) ppm.

## Refinement   

Crystal data, data collection and structure refinement details are summarized in Table 5 ▸. For both structures, the C-bound hydrogen atoms were positioned geometrically and refined using a riding model: C—H = 0.95–0.98 Å with *U*_iso_(H) = 1.5*U*_eq_(C-meth­yl) and 1.2*U*_iso_(C) for other H atoms.

## Supplementary Material

Crystal structure: contains datablock(s) ID1, ID2, Gobal. DOI: 10.1107/S205698901901329X/su5515sup1.cif

Structure factors: contains datablock(s) ID1. DOI: 10.1107/S205698901901329X/su5515ID1sup2.hkl

Structure factors: contains datablock(s) ID2. DOI: 10.1107/S205698901901329X/su5515ID2sup3.hkl

Supporting information file. DOI: 10.1107/S205698901901329X/su5515ID1sup4.cml

Supporting information file. DOI: 10.1107/S205698901901329X/su5515ID2sup5.cml

CCDC references: 1956419, 1956418, 1956418, 1956419

Additional supporting information:  crystallographic information; 3D view; checkCIF report

## Figures and Tables

**Table 1:** Dihedral angles between mol­ecular fragments (°) and mean deviations (Å) of atoms from these fragments

	ID[1]	ID[2]
Acceptor–bridge	1.31 (11)	11.6 (2)
Bridge–donor	2.63 (11)	4.9 (3)
		
Deviation in acceptor	0.0325	0.0159
Deviation in bridge	0.0000	0.0473
Deviation in donor	0.0035	0.0073

**Table 2:** Hydrogen-bond geometry (Å, °) for ID[1][Chem scheme1] *Cg*2 and *Cg*3 are the centroids of the C4–C9 and C10–C15 rings, respectively.

*D*—H⋯*A*	*D*—H	H⋯*A*	*D*⋯*A*	*D*—H⋯*A*
C14—H14⋯O1	0.95	2.17	3.0143 (12)	147
C7—H7⋯O3^i^	0.95	2.58	3.4813 (14)	159
C11—H11⋯O1^ii^	0.95	2.37	3.2778 (11)	160
C16—H16*A*⋯*Cg*3^iii^	0.98	2.91	3.7948 (12)	151
C17—H17*A*⋯*Cg*2^iv^	0.98	2.83	3.6591 (13)	143

Symmetry codes: (i) 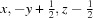; (ii) 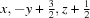; (iii) 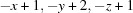; (iv) 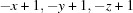.

**Table 3:** Hydrogen-bond geometry (Å, °) for ID[2][Chem scheme1]

*D*—H⋯*A*	*D*—H	H⋯*A*	*D*⋯*A*	*D*—H⋯*A*
C19—H19⋯O1	0.95	2.51	3.118 (3)	122
C17—H17*C*⋯O3^i^	0.98	2.50	3.463 (3)	169

Symmetry code: (i) 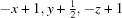.

**Table 4:** Absorption maxima (nm) for ID[1] and ID[2] in chloro­form and aceto­nitrile

Solvent	ID[1]	ID[2]
Chloro­form	483	539
Aceto­nitrile	480	526

**Table 5:** Experimental details

	ID[1]	ID[2]
Crystal data		
Chemical formula	C_18_H_15_NO_2_	C_20_H_17_NO_2_
*M*_r_	277.31	303.35
Crystal system, space group	Monoclinic, *P*2_1_/*c*	Monoclinic, *P*2_1_
Temperature (K)	150	150
*a*, *b*, *c* (Å)	9.2298 (9), 9.0302 (9), 16.7375 (17)	11.072 (2), 3.9664 (8), 17.557 (4)
β (°)	97.863 (1)	104.500 (2)
*V* (Å^3^)	1381.9 (2)	746.5 (3)
*Z*	4	2
Radiation type	Mo *K*α	Mo *K*α
μ (mm^−1^)	0.09	0.09
Crystal size (mm)	0.40 × 0.20 × 0.15	0.40 × 0.12 × 0.10

Data collection		
Diffractometer	Bruker APEXII CCD	Bruker APEXII CCD
Absorption correction	Multi-scan (*SADABS*; Krause *et al.*, 2015 ▸)	Multi-scan (*SADABS*; Krause *et al.*, 2015 ▸)
*T*_min_, *T*_max_	0.673, 0.746	0.584, 0.746
No. of measured, independent and observed [*I* > 2σ(*I*)] reflections	15794, 4391, 3812	8685, 4531, 4358
*R*_int_	0.041	0.044
(sin θ/λ)_max_ (Å^−1^)	0.741	0.739

Refinement		
*R*[*F* ^2^ > 2σ(*F* ^2^)], *wR*(*F* ^2^), *S*	0.047, 0.139, 1.07	0.044, 0.121, 1.12
No. of reflections	4391	4531
No. of parameters	192	210
No. of restraints	0	1
H-atom treatment	H-atom parameters constrained	H-atom parameters constrained
Δρ_max_, Δρ_min_ (e Å^−3^)	0.27, −0.40	0.22, −0.33
Absolute structure	–	Flack *x* determined using 1732 quotients [(*I* ^+^)−(*I* ^−^)]/[(*I* ^+^)+(*I* ^−^)] (Parsons *et al.*, 2013 ▸)
Absolute structure parameter	–	−0.1 (5)

Computer programs: *APEX3* and *SAINT* (Bruker, 2015 ▸), *SHELXT2017/1* (Sheldrick, 2015*a* ▸), *SHELXL2018/3* (Sheldrick, 2015*b* ▸), *Mercury* (Macrae *et al.*, 2008 ▸), *PLATON* (Spek, 2009 ▸) and *publCIF* (Westrip, 2010 ▸).

## supplementary crystallographic information

### 2-[4-(Dimethylamino)benzylidene]-1H-indene-1,3(2H)-dione (ID1) Crystal data

**Table:**

C_18_H_15_NO_2_	*F*(000) = 584
*M**_r_* = 277.31	*D*_x_ = 1.333 Mg m^−^^3^
Monoclinic, *P*2_1_/*c*	Mo *K*α radiation, λ = 0.71073 Å
*a* = 9.2298 (9) Å	Cell parameters from 8518 reflections
*b* = 9.0302 (9) Å	θ = 2.2–31.5°
*c* = 16.7375 (17) Å	µ = 0.09 mm^−^^1^
β = 97.863 (1)°	*T* = 150 K
*V* = 1381.9 (2) Å^3^	Block, purple
*Z* = 4	0.40 × 0.20 × 0.15 mm

### 2-[4-(Dimethylamino)benzylidene]-1H-indene-1,3(2H)-dione (ID1) Data collection

**Table:**

Bruker APEXII CCD diffractometer	3812 reflections with *I* > 2σ(*I*)
Radiation source: fine-focus sealed tube	*R*_int_ = 0.041
φ and ω scans	θ_max_ = 31.8°, θ_min_ = 2.2°
Absorption correction: multi-scan (SADABS; Krause *et al.*, 2015)	*h* = −13→12
*T*_min_ = 0.673, *T*_max_ = 0.746	*k* = −12→12
15794 measured reflections	*l* = −23→23
4391 independent reflections	

### 2-[4-(Dimethylamino)benzylidene]-1H-indene-1,3(2H)-dione (ID1) Refinement

**Table:**

Refinement on *F*^2^	Primary atom site location: dual
Least-squares matrix: full	Secondary atom site location: difference Fourier map
*R*[*F*^2^ > 2σ(*F*^2^)] = 0.047	Hydrogen site location: inferred from neighbouring sites
*wR*(*F*^2^) = 0.139	H-atom parameters constrained
*S* = 1.07	*w* = 1/[σ^2^(*F*_o_^2^) + (0.0835*P*)^2^ + 0.2073*P*] where *P* = (*F*_o_^2^ + 2*F*_c_^2^)/3
4391 reflections	(Δ/σ)_max_ < 0.001
192 parameters	Δρ_max_ = 0.27 e Å^−^^3^
0 restraints	Δρ_min_ = −0.40 e Å^−^^3^

### 2-[4-(Dimethylamino)benzylidene]-1H-indene-1,3(2H)-dione (ID1) Special details

**Table:**

Geometry. All esds (except the esd in the dihedral angle between two l.s. planes) are estimated using the full covariance matrix. The cell esds are taken into account individually in the estimation of esds in distances, angles and torsion angles; correlations between esds in cell parameters are only used when they are defined by crystal symmetry. An approximate (isotropic) treatment of cell esds is used for estimating esds involving l.s. planes.

### 2-[4-(Dimethylamino)benzylidene]-1H-indene-1,3(2H)-dione (ID1) Fractional atomic coordinates and isotropic or equivalent isotropic displacement parameters (Å^2^)

**Table:**

	*x*	*y*	*z*	*U*_iso_*/*U*_eq_	
O3	0.93689 (8)	0.32542 (8)	0.50819 (4)	0.03149 (17)	
O1	0.60774 (9)	0.60246 (9)	0.32291 (4)	0.0379 (2)	
N10	0.31173 (9)	0.97003 (9)	0.60039 (5)	0.02873 (18)	
C13	0.60837 (9)	0.65851 (10)	0.52818 (5)	0.02205 (17)	
C2	0.75196 (9)	0.49196 (9)	0.44122 (5)	0.02184 (17)	
C18	0.71343 (9)	0.55144 (10)	0.51060 (5)	0.02284 (17)	
H18	0.769381	0.512603	0.557817	0.027*	
C4	0.89040 (10)	0.33908 (10)	0.36262 (5)	0.02526 (18)	
C12	0.60627 (10)	0.69512 (10)	0.61024 (5)	0.02429 (18)	
H12	0.673418	0.647010	0.649979	0.029*	
C3	0.86960 (9)	0.37921 (10)	0.44692 (5)	0.02369 (18)	
C11	0.51125 (10)	0.79732 (10)	0.63494 (5)	0.02506 (18)	
H11	0.514728	0.819276	0.690677	0.030*	
C15	0.40888 (10)	0.83354 (10)	0.49501 (5)	0.02435 (18)	
H15	0.341038	0.880551	0.455185	0.029*	
C14	0.50520 (10)	0.73202 (10)	0.47141 (5)	0.02373 (18)	
H14	0.502565	0.710598	0.415658	0.028*	
C10	0.40801 (9)	0.87012 (9)	0.57750 (5)	0.02255 (17)	
C1	0.70060 (10)	0.51650 (10)	0.35504 (5)	0.02429 (18)	
C5	0.78862 (10)	0.41613 (10)	0.30928 (5)	0.02495 (18)	
C6	0.78078 (12)	0.39789 (12)	0.22630 (6)	0.0329 (2)	
H6	0.710143	0.449691	0.190125	0.039*	
C9	0.98955 (12)	0.24303 (13)	0.33465 (7)	0.0347 (2)	
H9	1.059397	0.190603	0.370999	0.042*	
C7	0.87982 (14)	0.30121 (14)	0.19785 (7)	0.0392 (3)	
H7	0.876810	0.286448	0.141453	0.047*	
C17	0.31815 (13)	1.01545 (13)	0.68433 (7)	0.0369 (2)	
H17A	0.297002	0.930205	0.717083	0.055*	
H17B	0.245649	1.093342	0.688545	0.055*	
H17C	0.416109	1.053349	0.703830	0.055*	
C16	0.19759 (12)	1.03450 (12)	0.54226 (7)	0.0367 (2)	
H16A	0.242318	1.089217	0.501365	0.055*	
H16B	0.138187	1.102198	0.570043	0.055*	
H16C	0.135358	0.955511	0.516197	0.055*	
C8	0.98325 (14)	0.22601 (14)	0.25148 (7)	0.0409 (3)	
H8	1.050850	0.161833	0.230903	0.049*	

### 2-[4-(Dimethylamino)benzylidene]-1H-indene-1,3(2H)-dione (ID1) Atomic displacement parameters (Å^2^)

**Table:**

	*U*^11^	*U*^22^	*U*^33^	*U*^12^	*U*^13^	*U*^23^
O3	0.0289 (3)	0.0367 (4)	0.0275 (3)	0.0027 (3)	−0.0011 (3)	0.0068 (3)
O1	0.0502 (5)	0.0424 (4)	0.0201 (3)	0.0190 (3)	0.0011 (3)	0.0033 (3)
N10	0.0287 (4)	0.0296 (4)	0.0275 (4)	0.0028 (3)	0.0026 (3)	−0.0008 (3)
C13	0.0230 (4)	0.0253 (4)	0.0174 (3)	−0.0038 (3)	0.0009 (3)	0.0006 (3)
C2	0.0224 (4)	0.0237 (4)	0.0190 (4)	−0.0023 (3)	0.0015 (3)	0.0023 (3)
C18	0.0233 (4)	0.0263 (4)	0.0183 (3)	−0.0032 (3)	0.0003 (3)	0.0023 (3)
C4	0.0244 (4)	0.0261 (4)	0.0258 (4)	−0.0015 (3)	0.0052 (3)	0.0014 (3)
C12	0.0254 (4)	0.0291 (4)	0.0174 (3)	−0.0012 (3)	−0.0007 (3)	0.0010 (3)
C3	0.0214 (4)	0.0257 (4)	0.0236 (4)	−0.0035 (3)	0.0017 (3)	0.0027 (3)
C11	0.0277 (4)	0.0288 (4)	0.0178 (3)	−0.0024 (3)	0.0003 (3)	−0.0015 (3)
C15	0.0246 (4)	0.0280 (4)	0.0194 (4)	−0.0021 (3)	−0.0008 (3)	0.0034 (3)
C14	0.0253 (4)	0.0281 (4)	0.0172 (3)	−0.0032 (3)	0.0008 (3)	0.0012 (3)
C10	0.0224 (4)	0.0221 (4)	0.0228 (4)	−0.0046 (3)	0.0017 (3)	0.0006 (3)
C1	0.0291 (4)	0.0251 (4)	0.0187 (4)	0.0003 (3)	0.0031 (3)	0.0013 (3)
C5	0.0277 (4)	0.0256 (4)	0.0218 (4)	−0.0005 (3)	0.0047 (3)	0.0006 (3)
C6	0.0401 (5)	0.0365 (5)	0.0224 (4)	0.0059 (4)	0.0056 (4)	0.0000 (4)
C9	0.0308 (5)	0.0378 (5)	0.0363 (5)	0.0076 (4)	0.0072 (4)	0.0026 (4)
C7	0.0470 (6)	0.0444 (6)	0.0283 (5)	0.0076 (5)	0.0126 (4)	−0.0028 (4)
C17	0.0393 (5)	0.0389 (5)	0.0333 (5)	0.0040 (4)	0.0079 (4)	−0.0076 (4)
C16	0.0306 (5)	0.0324 (5)	0.0449 (6)	0.0041 (4)	−0.0026 (4)	0.0035 (4)
C8	0.0427 (6)	0.0441 (6)	0.0385 (5)	0.0122 (5)	0.0145 (5)	−0.0021 (5)

### 2-[4-(Dimethylamino)benzylidene]-1H-indene-1,3(2H)-dione (ID1) Geometric parameters (Å, º)

**Table:**

O3—C3	1.2243 (11)	C15—C14	1.3722 (13)
O1—C1	1.2249 (11)	C15—C10	1.4207 (12)
N10—C10	1.3578 (12)	C15—H15	0.9500
N10—C16	1.4543 (13)	C14—H14	0.9500
N10—C17	1.4570 (13)	C1—C5	1.4961 (13)
C13—C14	1.4152 (12)	C5—C6	1.3907 (13)
C13—C12	1.4155 (12)	C6—C7	1.3941 (15)
C13—C18	1.4281 (12)	C6—H6	0.9500
C2—C18	1.3700 (12)	C9—C8	1.3939 (16)
C2—C1	1.4721 (11)	C9—H9	0.9500
C2—C3	1.4820 (12)	C7—C8	1.3941 (17)
C18—H18	0.9500	C7—H7	0.9500
C4—C9	1.3879 (13)	C17—H17A	0.9800
C4—C5	1.3907 (13)	C17—H17B	0.9800
C4—C3	1.4943 (13)	C17—H17C	0.9800
C12—C11	1.3751 (13)	C16—H16A	0.9800
C12—H12	0.9500	C16—H16B	0.9800
C11—C10	1.4190 (12)	C16—H16C	0.9800
C11—H11	0.9500	C8—H8	0.9500
			
C10—N10—C16	121.29 (9)	C11—C10—C15	117.27 (8)
C10—N10—C17	121.15 (8)	O1—C1—C2	129.63 (8)
C16—N10—C17	117.56 (9)	O1—C1—C5	123.74 (8)
C14—C13—C12	116.40 (8)	C2—C1—C5	106.61 (7)
C14—C13—C18	126.35 (8)	C6—C5—C4	121.34 (9)
C12—C13—C18	117.25 (8)	C6—C5—C1	128.71 (8)
C18—C2—C1	133.27 (8)	C4—C5—C1	109.93 (8)
C18—C2—C3	119.19 (8)	C5—C6—C7	117.92 (9)
C1—C2—C3	107.54 (7)	C5—C6—H6	121.0
C2—C18—C13	134.62 (8)	C7—C6—H6	121.0
C2—C18—H18	112.7	C4—C9—C8	117.80 (10)
C13—C18—H18	112.7	C4—C9—H9	121.1
C9—C4—C5	120.99 (9)	C8—C9—H9	121.1
C9—C4—C3	130.22 (9)	C6—C7—C8	120.56 (10)
C5—C4—C3	108.79 (8)	C6—C7—H7	119.7
C11—C12—C13	122.85 (8)	C8—C7—H7	119.7
C11—C12—H12	118.6	N10—C17—H17A	109.5
C13—C12—H12	118.6	N10—C17—H17B	109.5
O3—C3—C2	127.57 (8)	H17A—C17—H17B	109.5
O3—C3—C4	125.36 (9)	N10—C17—H17C	109.5
C2—C3—C4	107.06 (7)	H17A—C17—H17C	109.5
C12—C11—C10	120.27 (8)	H17B—C17—H17C	109.5
C12—C11—H11	119.9	N10—C16—H16A	109.5
C10—C11—H11	119.9	N10—C16—H16B	109.5
C14—C15—C10	121.68 (8)	H16A—C16—H16B	109.5
C14—C15—H15	119.2	N10—C16—H16C	109.5
C10—C15—H15	119.2	H16A—C16—H16C	109.5
C15—C14—C13	121.53 (8)	H16B—C16—H16C	109.5
C15—C14—H14	119.2	C9—C8—C7	121.37 (10)
C13—C14—H14	119.2	C9—C8—H8	119.3
N10—C10—C11	121.43 (8)	C7—C8—H8	119.3
N10—C10—C15	121.31 (8)		
			
C1—C2—C18—C13	0.93 (17)	C12—C11—C10—C15	−0.50 (13)
C3—C2—C18—C13	−179.71 (9)	C14—C15—C10—N10	−179.59 (8)
C14—C13—C18—C2	2.59 (16)	C14—C15—C10—C11	0.04 (13)
C12—C13—C18—C2	−177.54 (9)	C18—C2—C1—O1	1.45 (18)
C14—C13—C12—C11	−0.66 (13)	C3—C2—C1—O1	−177.97 (10)
C18—C13—C12—C11	179.46 (8)	C18—C2—C1—C5	179.75 (9)
C18—C2—C3—O3	2.96 (14)	C3—C2—C1—C5	0.33 (9)
C1—C2—C3—O3	−177.53 (9)	C9—C4—C5—C6	1.13 (15)
C18—C2—C3—C4	−178.21 (8)	C3—C4—C5—C6	−178.70 (9)
C1—C2—C3—C4	1.30 (9)	C9—C4—C5—C1	−177.34 (9)
C9—C4—C3—O3	−3.52 (16)	C3—C4—C5—C1	2.83 (10)
C5—C4—C3—O3	176.28 (9)	O1—C1—C5—C6	−1.89 (16)
C9—C4—C3—C2	177.62 (10)	C2—C1—C5—C6	179.68 (10)
C5—C4—C3—C2	−2.58 (10)	O1—C1—C5—C4	176.43 (9)
C13—C12—C11—C10	0.83 (14)	C2—C1—C5—C4	−2.00 (10)
C10—C15—C14—C13	0.11 (13)	C4—C5—C6—C7	−0.91 (16)
C12—C13—C14—C15	0.18 (13)	C1—C5—C6—C7	177.25 (10)
C18—C13—C14—C15	−179.95 (8)	C5—C4—C9—C8	−0.25 (16)
C16—N10—C10—C11	−174.40 (9)	C3—C4—C9—C8	179.53 (10)
C17—N10—C10—C11	4.97 (14)	C5—C6—C7—C8	−0.15 (18)
C16—N10—C10—C15	5.21 (14)	C4—C9—C8—C7	−0.81 (18)
C17—N10—C10—C15	−175.41 (9)	C6—C7—C8—C9	1.0 (2)
C12—C11—C10—N10	179.13 (8)		

### 2-[4-(Dimethylamino)benzylidene]-1H-indene-1,3(2H)-dione (ID1) Hydrogen-bond geometry (Å, º)

*Cg*2 and *Cg*3 are the centroids of the C4–C9 and C10–C15 rings, respectively.

**Table:**

*D*—H···*A*	*D*—H	H···*A*	*D*···*A*	*D*—H···*A*
C14—H14···O1	0.95	2.17	3.0143 (12)	147
C7—H7···O3^i^	0.95	2.58	3.4813 (14)	159
C11—H11···O1^ii^	0.95	2.37	3.2778 (11)	160
C16—H16*A*···*Cg*3^iii^	0.98	2.91	3.7948 (12)	151
C17—H17*A*···*Cg*2^iv^	0.98	2.83	3.6591 (13)	143

Symmetry codes: (i) *x*, −*y*+1/2, *z*−1/2; (ii) *x*, −*y*+3/2, *z*+1/2; (iii) −*x*+1, −*y*+2, −*z*+1; (iv) −*x*+1, −*y*+1, −*z*+1.

### (E)-2-{3-[4-(Dimethylamino)phenyl]allylidene}-1H-indene-1,3(2H)-dione (ID2) Crystal data

**Table:**

C_20_H_17_NO_2_	*F*(000) = 320
*M**_r_* = 303.35	*D*_x_ = 1.350 Mg m^−^^3^
Monoclinic, *P*2_1_	Mo *K*α radiation, λ = 0.71073 Å
*a* = 11.072 (2) Å	Cell parameters from 6074 reflections
*b* = 3.9664 (8) Å	θ = 2.4–31.6°
*c* = 17.557 (4) Å	µ = 0.09 mm^−^^1^
β = 104.500 (2)°	*T* = 150 K
*V* = 746.5 (3) Å^3^	Needle, blue
*Z* = 2	0.40 × 0.12 × 0.10 mm

### (E)-2-{3-[4-(Dimethylamino)phenyl]allylidene}-1H-indene-1,3(2H)-dione (ID2) Data collection

**Table:**

Bruker APEXII CCD diffractometer	4358 reflections with *I* > 2σ(*I*)
Radiation source: fine-focus sealed tube	*R*_int_ = 0.044
φ and ω scans	θ_max_ = 31.7°, θ_min_ = 1.2°
Absorption correction: multi-scan (SADABS; Krause *et al.*, 2015)	*h* = −15→16
*T*_min_ = 0.584, *T*_max_ = 0.746	*k* = −5→5
8685 measured reflections	*l* = −24→25
4531 independent reflections	

### (E)-2-{3-[4-(Dimethylamino)phenyl]allylidene}-1H-indene-1,3(2H)-dione (ID2) Refinement

**Table:**

Refinement on *F*^2^	Secondary atom site location: difference Fourier map
Least-squares matrix: full	Hydrogen site location: inferred from neighbouring sites
*R*[*F*^2^ > 2σ(*F*^2^)] = 0.044	H-atom parameters constrained
*wR*(*F*^2^) = 0.121	*w* = 1/[σ^2^(*F*_o_^2^) + (0.0488*P*)^2^ + 0.2055*P*] where *P* = (*F*_o_^2^ + 2*F*_c_^2^)/3
*S* = 1.12	(Δ/σ)_max_ < 0.001
4531 reflections	Δρ_max_ = 0.22 e Å^−^^3^
210 parameters	Δρ_min_ = −0.33 e Å^−^^3^
1 restraint	Absolute structure: Flack *x* determined using 1732 quotients [(*I*^+^)-(*I*^-^)]/[(*I*^+^)+(*I*^-^)] (Parsons *et al.*, 2013)
Primary atom site location: dual	Absolute structure parameter: −0.1 (5)

### (E)-2-{3-[4-(Dimethylamino)phenyl]allylidene}-1H-indene-1,3(2H)-dione (ID2) Special details

**Table:**

Geometry. All esds (except the esd in the dihedral angle between two l.s. planes) are estimated using the full covariance matrix. The cell esds are taken into account individually in the estimation of esds in distances, angles and torsion angles; correlations between esds in cell parameters are only used when they are defined by crystal symmetry. An approximate (isotropic) treatment of cell esds is used for estimating esds involving l.s. planes.

### (E)-2-{3-[4-(Dimethylamino)phenyl]allylidene}-1H-indene-1,3(2H)-dione (ID2) Fractional atomic coordinates and isotropic or equivalent isotropic displacement parameters (Å^2^)

**Table:**

	*x*	*y*	*z*	*U*_iso_*/*U*_eq_	
O3	0.50946 (14)	0.2510 (5)	0.17850 (9)	0.0331 (4)	
O1	0.11093 (14)	0.5986 (5)	0.20367 (9)	0.0328 (4)	
N10	0.15603 (16)	0.3906 (5)	0.69990 (9)	0.0253 (4)	
C13	0.29025 (17)	0.2553 (5)	0.49784 (10)	0.0203 (3)	
C10	0.20054 (16)	0.3470 (5)	0.63500 (10)	0.0202 (3)	
C12	0.35740 (17)	0.1388 (5)	0.57237 (11)	0.0232 (4)	
H12	0.434333	0.025205	0.576557	0.028*	
C9	0.37083 (18)	0.5984 (6)	0.02319 (11)	0.0250 (4)	
H9	0.450310	0.528451	0.017745	0.030*	
C11	0.31564 (18)	0.1834 (5)	0.63920 (11)	0.0236 (4)	
H11	0.364293	0.103946	0.688397	0.028*	
C7	0.16966 (19)	0.8643 (6)	−0.03035 (11)	0.0276 (4)	
H7	0.113386	0.972479	−0.073153	0.033*	
C14	0.17745 (18)	0.4244 (5)	0.49465 (11)	0.0224 (4)	
H14	0.130688	0.510783	0.445647	0.027*	
C19	0.29104 (17)	0.3097 (5)	0.35537 (11)	0.0230 (4)	
H19	0.211433	0.414940	0.342126	0.028*	
C18	0.35755 (17)	0.2675 (5)	0.29653 (10)	0.0222 (3)	
H18	0.433577	0.144955	0.311130	0.027*	
C20	0.33964 (17)	0.2017 (5)	0.43054 (11)	0.0222 (4)	
H20	0.414971	0.076184	0.439446	0.027*	
C17	0.2246 (2)	0.2557 (6)	0.77578 (11)	0.0294 (4)	
H17A	0.236951	0.012715	0.770933	0.044*	
H17B	0.176965	0.296073	0.815026	0.044*	
H17C	0.305856	0.367609	0.792316	0.044*	
C8	0.2871 (2)	0.7601 (6)	−0.03803 (11)	0.0273 (4)	
H8	0.309669	0.800923	−0.085963	0.033*	
C6	0.13502 (17)	0.8102 (5)	0.03978 (11)	0.0247 (4)	
H6	0.056140	0.882629	0.045770	0.030*	
C16	0.03720 (19)	0.5566 (6)	0.69463 (13)	0.0283 (4)	
H16A	0.040550	0.787428	0.675361	0.043*	
H16B	0.020234	0.562374	0.746783	0.043*	
H16C	−0.029316	0.431904	0.658184	0.043*	
C4	0.33549 (16)	0.5416 (5)	0.09239 (10)	0.0200 (3)	
C15	0.13295 (17)	0.4683 (5)	0.56017 (11)	0.0224 (4)	
H15	0.055820	0.581423	0.555547	0.027*	
C3	0.40481 (17)	0.3712 (5)	0.16627 (10)	0.0216 (3)	
C5	0.21913 (16)	0.6474 (5)	0.10052 (10)	0.0195 (3)	
C2	0.32319 (17)	0.3854 (5)	0.22113 (10)	0.0212 (3)	
C1	0.20501 (17)	0.5508 (5)	0.18022 (11)	0.0219 (3)	

### (E)-2-{3-[4-(Dimethylamino)phenyl]allylidene}-1H-indene-1,3(2H)-dione (ID2) Atomic displacement parameters (Å^2^)

**Table:**

	*U*^11^	*U*^22^	*U*^33^	*U*^12^	*U*^13^	*U*^23^
O3	0.0219 (6)	0.0463 (10)	0.0311 (7)	0.0089 (7)	0.0068 (5)	0.0039 (7)
O1	0.0235 (6)	0.0485 (10)	0.0297 (7)	0.0070 (7)	0.0128 (5)	0.0051 (7)
N10	0.0264 (8)	0.0304 (9)	0.0201 (7)	0.0016 (7)	0.0078 (6)	0.0028 (7)
C13	0.0197 (7)	0.0221 (8)	0.0182 (7)	−0.0011 (7)	0.0029 (6)	0.0026 (6)
C10	0.0196 (7)	0.0211 (8)	0.0190 (7)	−0.0023 (7)	0.0035 (6)	0.0013 (6)
C12	0.0187 (7)	0.0278 (9)	0.0219 (8)	0.0035 (7)	0.0028 (6)	0.0038 (7)
C9	0.0241 (8)	0.0292 (10)	0.0240 (8)	−0.0017 (8)	0.0103 (7)	−0.0015 (7)
C11	0.0214 (8)	0.0286 (10)	0.0189 (7)	0.0020 (7)	0.0011 (6)	0.0041 (7)
C7	0.0290 (9)	0.0305 (10)	0.0208 (8)	−0.0019 (8)	0.0017 (7)	0.0038 (8)
C14	0.0203 (8)	0.0245 (9)	0.0198 (7)	−0.0002 (7)	0.0003 (6)	0.0041 (7)
C19	0.0212 (8)	0.0267 (9)	0.0206 (7)	−0.0009 (7)	0.0046 (6)	0.0010 (7)
C18	0.0217 (8)	0.0237 (9)	0.0206 (7)	−0.0007 (7)	0.0038 (6)	−0.0001 (7)
C20	0.0208 (8)	0.0242 (9)	0.0213 (8)	−0.0009 (7)	0.0046 (6)	0.0008 (7)
C17	0.0351 (10)	0.0348 (11)	0.0176 (8)	−0.0032 (9)	0.0051 (7)	0.0012 (8)
C8	0.0315 (9)	0.0313 (10)	0.0198 (8)	−0.0033 (8)	0.0076 (7)	0.0005 (7)
C6	0.0198 (8)	0.0288 (10)	0.0243 (8)	−0.0002 (7)	0.0035 (6)	0.0026 (7)
C16	0.0257 (9)	0.0299 (10)	0.0320 (10)	−0.0005 (8)	0.0121 (7)	−0.0022 (8)
C4	0.0186 (7)	0.0222 (8)	0.0191 (7)	−0.0014 (7)	0.0047 (6)	−0.0026 (7)
C15	0.0185 (8)	0.0261 (9)	0.0206 (8)	0.0021 (7)	0.0012 (6)	0.0032 (7)
C3	0.0187 (7)	0.0255 (9)	0.0206 (7)	0.0003 (7)	0.0045 (6)	−0.0011 (7)
C5	0.0173 (7)	0.0221 (8)	0.0186 (7)	−0.0013 (6)	0.0037 (6)	−0.0010 (6)
C2	0.0189 (7)	0.0254 (9)	0.0190 (7)	0.0001 (7)	0.0042 (6)	−0.0007 (7)
C1	0.0195 (7)	0.0266 (9)	0.0201 (7)	−0.0001 (7)	0.0059 (6)	0.0002 (7)

### (E)-2-{3-[4-(Dimethylamino)phenyl]allylidene}-1H-indene-1,3(2H)-dione (ID2) Geometric parameters (Å, º)

**Table:**

O3—C3	1.221 (2)	C19—C20	1.364 (3)
O1—C1	1.227 (2)	C19—C18	1.421 (2)
N10—C10	1.361 (2)	C19—H19	0.9500
N10—C16	1.453 (3)	C18—C2	1.365 (3)
N10—C17	1.460 (3)	C18—H18	0.9500
C13—C14	1.406 (3)	C20—H20	0.9500
C13—C12	1.411 (2)	C17—H17A	0.9800
C13—C20	1.437 (2)	C17—H17B	0.9800
C10—C11	1.416 (3)	C17—H17C	0.9800
C10—C15	1.423 (2)	C8—H8	0.9500
C12—C11	1.376 (3)	C6—C5	1.387 (3)
C12—H12	0.9500	C6—H6	0.9500
C9—C4	1.385 (2)	C16—H16A	0.9800
C9—C8	1.389 (3)	C16—H16B	0.9800
C9—H9	0.9500	C16—H16C	0.9800
C11—H11	0.9500	C4—C5	1.396 (2)
C7—C6	1.395 (3)	C4—C3	1.493 (3)
C7—C8	1.402 (3)	C15—H15	0.9500
C7—H7	0.9500	C3—C2	1.478 (2)
C14—C15	1.371 (3)	C5—C1	1.496 (2)
C14—H14	0.9500	C2—C1	1.479 (3)
			
C10—N10—C16	121.07 (16)	H17A—C17—H17B	109.5
C10—N10—C17	120.19 (17)	N10—C17—H17C	109.5
C16—N10—C17	118.67 (16)	H17A—C17—H17C	109.5
C14—C13—C12	116.72 (16)	H17B—C17—H17C	109.5
C14—C13—C20	123.71 (16)	C9—C8—C7	121.16 (18)
C12—C13—C20	119.57 (17)	C9—C8—H8	119.4
N10—C10—C11	121.65 (16)	C7—C8—H8	119.4
N10—C10—C15	120.86 (17)	C5—C6—C7	118.06 (18)
C11—C10—C15	117.49 (16)	C5—C6—H6	121.0
C11—C12—C13	122.48 (17)	C7—C6—H6	121.0
C11—C12—H12	118.8	N10—C16—H16A	109.5
C13—C12—H12	118.8	N10—C16—H16B	109.5
C4—C9—C8	118.26 (18)	H16A—C16—H16B	109.5
C4—C9—H9	120.9	N10—C16—H16C	109.5
C8—C9—H9	120.9	H16A—C16—H16C	109.5
C12—C11—C10	120.33 (16)	H16B—C16—H16C	109.5
C12—C11—H11	119.8	C9—C4—C5	120.77 (17)
C10—C11—H11	119.8	C9—C4—C3	129.72 (17)
C6—C7—C8	120.39 (18)	C5—C4—C3	109.52 (15)
C6—C7—H7	119.8	C14—C15—C10	121.01 (17)
C8—C7—H7	119.8	C14—C15—H15	119.5
C15—C14—C13	121.95 (17)	C10—C15—H15	119.5
C15—C14—H14	119.0	O3—C3—C2	127.48 (18)
C13—C14—H14	119.0	O3—C3—C4	126.02 (17)
C20—C19—C18	121.06 (18)	C2—C3—C4	106.49 (15)
C20—C19—H19	119.5	C6—C5—C4	121.36 (16)
C18—C19—H19	119.5	C6—C5—C1	129.12 (16)
C2—C18—C19	126.54 (18)	C4—C5—C1	109.51 (15)
C2—C18—H18	116.7	C18—C2—C3	123.48 (17)
C19—C18—H18	116.7	C18—C2—C1	128.36 (17)
C19—C20—C13	127.60 (18)	C3—C2—C1	108.14 (15)
C19—C20—H20	116.2	O1—C1—C2	128.88 (18)
C13—C20—H20	116.2	O1—C1—C5	124.79 (17)
N10—C17—H17A	109.5	C2—C1—C5	106.31 (15)
N10—C17—H17B	109.5		
			
C16—N10—C10—C11	−179.48 (19)	C5—C4—C3—O3	−179.7 (2)
C17—N10—C10—C11	−2.5 (3)	C9—C4—C3—C2	179.4 (2)
C16—N10—C10—C15	1.1 (3)	C5—C4—C3—C2	−1.0 (2)
C17—N10—C10—C15	178.13 (19)	C7—C6—C5—C4	0.3 (3)
C14—C13—C12—C11	0.5 (3)	C7—C6—C5—C1	−178.4 (2)
C20—C13—C12—C11	179.7 (2)	C9—C4—C5—C6	0.4 (3)
C13—C12—C11—C10	0.9 (3)	C3—C4—C5—C6	−179.24 (18)
N10—C10—C11—C12	179.1 (2)	C9—C4—C5—C1	179.36 (18)
C15—C10—C11—C12	−1.5 (3)	C3—C4—C5—C1	−0.3 (2)
C12—C13—C14—C15	−1.4 (3)	C19—C18—C2—C3	171.6 (2)
C20—C13—C14—C15	179.5 (2)	C19—C18—C2—C1	−6.9 (4)
C20—C19—C18—C2	−175.0 (2)	O3—C3—C2—C18	1.7 (3)
C18—C19—C20—C13	173.3 (2)	C4—C3—C2—C18	−176.93 (19)
C14—C13—C20—C19	2.4 (3)	O3—C3—C2—C1	−179.5 (2)
C12—C13—C20—C19	−176.7 (2)	C4—C3—C2—C1	1.9 (2)
C4—C9—C8—C7	0.1 (3)	C18—C2—C1—O1	−4.8 (4)
C6—C7—C8—C9	0.7 (3)	C3—C2—C1—O1	176.5 (2)
C8—C7—C6—C5	−0.9 (3)	C18—C2—C1—C5	176.7 (2)
C8—C9—C4—C5	−0.6 (3)	C3—C2—C1—C5	−2.0 (2)
C8—C9—C4—C3	179.0 (2)	C6—C5—C1—O1	1.7 (3)
C13—C14—C15—C10	0.8 (3)	C4—C5—C1—O1	−177.2 (2)
N10—C10—C15—C14	−179.92 (19)	C6—C5—C1—C2	−179.7 (2)
C11—C10—C15—C14	0.7 (3)	C4—C5—C1—C2	1.4 (2)
C9—C4—C3—O3	0.7 (4)		

### (E)-2-{3-[4-(Dimethylamino)phenyl]allylidene}-1H-indene-1,3(2H)-dione (ID2) Hydrogen-bond geometry (Å, º)

**Table:**

*D*—H···*A*	*D*—H	H···*A*	*D*···*A*	*D*—H···*A*
C19—H19···O1	0.95	2.51	3.118 (3)	122
C17—H17*C*···O3^i^	0.98	2.50	3.463 (3)	169

Symmetry code: (i) −*x*+1, *y*+1/2, −*z*+1.
